# Intensive Care Unit Physicians’ Perspectives on Artificial Intelligence–Based Clinical Decision Support Tools: Preimplementation Survey Study

**DOI:** 10.2196/39114

**Published:** 2023-01-05

**Authors:** Siri L van der Meijden, Anne A H de Hond, Patrick J Thoral, Ewout W Steyerberg, Ilse M J Kant, Giovanni Cinà, M Sesmu Arbous

**Affiliations:** 1 Department of Intensive Care Medicine Leiden University Medical Center Leiden Netherlands; 2 Clinical AI Implementation and Research Lab Leiden University Medical Center Leiden Netherlands; 3 Healthplus.ai Amsterdam Netherlands; 4 Department of Biomedical Data Sciences Leiden University Medical Center Leiden Netherlands; 5 Department of Intensive Care Medicine, Laboratory for Critical Care Computational Intelligence, Amsterdam Medical Data Science Amsterdam University Medical Centers Amsterdam Netherlands; 6 Pacmed Amsterdam Netherlands; 7 Institute for Logic, Language and Computation University of Amsterdam Amsterdam Netherlands; 8 Department of Medical Informatics Amsterdam University Medical Center University of Amsterdam Amsterdam Netherlands

**Keywords:** intensive care unit, hospital, discharge, artificial intelligence, AI, clinical decision support, clinical support, acceptance, decision support, decision-making, digital health, eHealth, survey, perspective, attitude, opinion, adoption, prediction, risk

## Abstract

**Background:**

Artificial intelligence–based clinical decision support (AI-CDS) tools have great potential to benefit intensive care unit (ICU) patients and physicians. There is a gap between the development and implementation of these tools.

**Objective:**

We aimed to investigate physicians’ perspectives and their current decision-making behavior before implementing a discharge AI-CDS tool for predicting readmission and mortality risk after ICU discharge.

**Methods:**

We conducted a survey of physicians involved in decision-making on discharge of patients at two Dutch academic ICUs between July and November 2021. Questions were divided into four domains: (1) physicians’ current decision-making behavior with respect to discharging ICU patients, (2) perspectives on the use of AI-CDS tools in general, (3) willingness to incorporate a discharge AI-CDS tool into daily clinical practice, and (4) preferences for using a discharge AI-CDS tool in daily workflows.

**Results:**

Most of the 64 respondents (of 93 contacted, 69%) were familiar with AI (62/64, 97%) and had positive expectations of AI, with 55 of 64 (86%) believing that AI could support them in their work as a physician. The respondents disagreed on whether the decision to discharge a patient was complex (23/64, 36% agreed and 22/64, 34% disagreed); nonetheless, most (59/64, 92%) agreed that a discharge AI-CDS tool could be of value. Significant differences were observed between physicians from the 2 academic sites, which may be related to different levels of involvement in the development of the discharge AI-CDS tool.

**Conclusions:**

ICU physicians showed a favorable attitude toward the integration of AI-CDS tools into the ICU setting in general, and in particular toward a tool to predict a patient’s risk of readmission and mortality within 7 days after discharge. The findings of this questionnaire will be used to improve the implementation process and training of end users.

## Introduction

Due to the increasing availability of high-quality clinical data, the development of artificial intelligence–based clinical decision support (AI-CDS) tools to enhance personalized medicine is on the rise. AI-CDS tools make use of learning algorithms, including machine learning, which may, in specific circumstances, outperform classical statistical models when applied to large data sets for health care–related prediction tasks [1-3]. The complex nature of these artificial intelligence (AI) algorithms and their use of numerous input variables may lead to “black box” algorithms, which often leave it unclear why the algorithm output specific predictions [4,5]. In the intensive care unit (ICU), complex and high-stakes decisions are made that might benefit from data-driven decision support [6]. The ICU is the most data-rich environment in the hospital due to high-frequency monitoring, and there has been an increase in the literature on AI model development for ICU decision support [7]. However, a recent review showed that implementation of these AI-CDS tools in clinical ICU practice is lacking due to difficulties at several levels [8]. These difficulties include patient privacy, regulatory aspects, and a lack of demonstrations of these tools’ clinical value in the complex ICU environment [8]. To enhance clinical uptake and integration in daily workflows and to tailor AI-CDS tools to physicians’ needs, we need a broad understanding of physicians’ current decision-making practices and their views on the use of AI-CDS tools [9-11].

There is a need to study human factors for the safe and effective implementation of AI-CDS tools, as high predictive performance does not ensure acceptance of these technologies [12,13]. Physicians’ perspectives on clinical AI have been investigated in survey studies in the fields of psychiatry [14], gastroenterology [15], diagnostic pathology [16], and cardiology [17], as well as across specialties [18-20]. In general, strong interest and favorable attitudes toward the use of AI-CDS were reported, but no study has focused solely on the application of AI-CDS tools in the ICU in terms of willingness to use such a tool in clinical practice and how it would fit into clinical workflows. As the ICU is unique in terms of the complexity of decisions, the pressure under which decisions have to be made, and the potential in terms of data availability, knowledge focused on this clinical domain is highly relevant. To understand the potential of AI-CDS tools in the data-rich ICU environment, and to attempt to solve the challenging “last mile” problem facing real-world implementations, we need to gather more insights on clinicians’ attitudes and perspectives regarding this subject in the local context [21,22]. These insights may enhance successful implementation in this high-stakes decision-making environment, as clinician input is important throughout the implementation process to enhance successful implementation and ultimately improve patient outcomes [23].

This survey study is part of preimplementation research for Pacmed Critical [24]. Pacmed Critical is a machine learning–based AI-CDS tool that predicts a patient’s combined readmission and mortality risk within 7 days of ICU discharge to support physicians in their decisions to discharge patients to lower care wards [25,26]. The Pacmed Critical software is intended for use as a complementary tool by qualified ICU medical professionals and will be accessed on hospital premises; it will not be used on mobile devices. We aimed to investigate (1) physicians’ current decision-making behavior with respect to discharging ICU patients, (2) physicians’ perspectives on the use of AI decision support tools in general, (3) physicians’ willingness to incorporate an AI-CDS tool in daily clinical practice, and (4) physicians’ preferences for using an AI-CDS tool in their daily workflows. As knowledge of physicians’ attitudes toward the implementation of AI-CDS tools is currently lacking for the ICU domain, the overall aim of this survey was to investigate ICU physicians’ perspectives on AI-CDS tools to enhance the implementation process and to raise awareness among ICU physicians of an upcoming implementation.

## Methods

### Study Sample

The survey was conducted between July and December 2021 at Leiden University Medical Center (LUMC), Leiden, and Amsterdam University Medical Center (Amsterdam UMC, Vrije Universiteit Medical Center location), Amsterdam, both in the Netherlands. Both centers are academic tertiary referral hospitals. At Amsterdam UMC, 2 intensivists codeveloped Pacmed Critical, and other ICU clinicians took part in end user testing as part of the Conformité Européene (CE) certification process of the software. The LUMC physicians were not involved in the development of the tool, and implementation was planned to start after completion of the survey.

Results were collected anonymously on paper at LUMC and by means of a web-based survey at Amsterdam UMC. All physicians working at the ICU were eligible to participate in this study, including residents, intensive care fellows, and board-certified intensivists.

### Ethics Approval

The results of this research do not include any sensitive or identifiable data. We obtained ethical approval from the medical ethical committee of LUMC (ID: N21.153).

### Survey Instrument

The survey instrument was developed with the expertise of 2 ICU physicians, AI and organizational researchers, a data scientist, a user experience researcher, and a Pacmed Critical product owner. The 20-question survey consisted of 13 statements, 5 multiple-choice questions, and 2 open questions. Statements were answered on a 5-point Likert scale ranging from 1 (strongly disagree) to 5 (strongly agree) [27]. Multimedia Appendices 1 and 2 show the full questionnaire (in English and Dutch, respectively); Multimedia Appendix 1 also describes the rationale for each survey question. Participants did not receive additional background information on Pacmed Critical other than that it used an AI algorithm based on patient data from electronic health records (EHRs). The survey was divided into 4 domains. These 4 domains and individual questions in the domains were chosen to obtain knowledge to optimize further development and enhance the implementation process.

#### Physicians’ Current Decision-Making Behavior With Respect to Discharging ICU Patients (Q1-3, Q11-13)

The aim of the questions in this domain was to investigate current decision-making and whether the discharge AI-CDS tool could be of benefit in terms of the complexity of the discharge decision and the predicted outcome. The first 3 statement questions investigated the complexity of the decision to discharge ICU patients and the influence of readmission risk and bed availability on this decision. The average certainty that a patient would not be readmitted after the decision to discharge was made was ranked on a scale from 1 (completely uncertain) to 10 (completely certain). We asked about patient groups for whom the decision to discharge was perceived as most challenging to determine where the AI-CDS tool could be of most value (these questions were multiple choice). We also asked about which factors were deemed most important in the process of discharging patients (open answers were solicited).

#### Perspectives on the Use of AI-CDS Tools in General (Q4-8)

Five statements covered perspectives and attitudes toward AI-CDS tools at the ICU, as the participants had no or little experience in working with these tools. These included statements on familiarity with AI, whether AI was believed to be able to replace physicians in the future, the anticipated added value and support of AI-CDS at the ICU, and whether AI-CDS tools represented the physicians’ work sufficiently to be of support.

#### Willingness to Incorporate the Discharge AI-CDS Tool Into Daily Clinical Practice (Q9,10, 17-20)

The willingness to incorporate the discharge AI-CDS in clinical practice was assessed with 5 statements on belief in the positive value of discharge decision support, the importance of having insight into the contributing factors to the prediction, the potential influence a prediction may have on discharge decision-making, willingness to consult the prediction before making the decision to discharge a patient, and the feasibility of incorporating the prediction into the physicians’ workflows. Furthermore, the physicians were asked to indicate the threshold of predicted readmission and mortality risk (on a scale of 0 to 100) above which they would not discharge a patient to the ward, and below which they would discharge a patient. The aim of this question was to study the influence of a certain predicted chance of readmission and mortality on the physician’s behavior.

#### Preferences for Using a Discharge AI-CDS tool in Daily Workflows (Q14-16)

The last domain included questions on how the AI-CDS tool for discharging ICU patients could be integrated into the current clinical workflow at the ICU; the answers were intended to be used as input in the design and implementation process of the AI-CDS tool, in order to make it part of current decision-making processes [11]. We used multiple-choice questions to determine the preferred method to access the predictions (ie, on a dashboard or integrated in EHRs), the preferred moment or moments to access the predictions, when the predictions should be updated, and the most relevant end users. Information gathered from these questions was used to understand the demands on the user interface and to optimize implementation and daily use. One or more options could be chosen for the multiple-choice questions. Lastly, respondents could leave open comments and suggestions.

### Data Analysis

Results are given as percentages of the total number of respondents for categorical questions. Answers to numerical questions are summarized as the median (IQR). Because the participating physicians at the 2 centers differed in their involvement in the development of the tool, we performed separate analyses for LUMC and Amsterdam UMC for the questions in domain 1 (current decision-making behavior with respect to discharging ICU patients), domain 2 (attitudes and perspectives on AI-CDS), and domain 3 (willingness to incorporate a discharge AI-CDS tool into daily clinical practice). As an additional subgroup analysis, we investigated differences in the responses to the questions in domains 2 and 3 between intensivists and other physicians working at the ICU (ie, residents and fellows at the ICU). We determined significant associations for the Likert-scale statement questions with the Mann-Whitney *U* test. The level of significance was set at *P*<.05.

## Results

### Sample Characteristics

The survey was distributed to 40 clinicians at LUMC and 53 clinicians at Amsterdam UMC. A total of 64 of 93 (69%) of these clinicians completed the survey, including 33 of 64 (52%) at LUMC and 31 of 64 (48%) at Amsterdam UMC (Table 1). The total group had a median 2.75 (IQR 1-10) years of ICU work experience; the LUMC group had 3 (IQR 1-10.5) years and the Amsterdam UMC group 2 (IQR 1.5-10) years (*P*=.94). In the Netherlands, medical residents from many specialties are assigned a rotation in the ICU as part of their specialist training, which is reflected by the variety of medical specialists represented in the survey (Table 1).

**Table 1:** Response rate, level of training, and medical specialties of respondents. Probabilities may not add up to 100% due to rounding.

			Leiden University Medical Center (n=33), n (%)^a^		Amsterdam University Medical Center (n=31), n (%)^b^		Total (N=64), n (%)
**Level of training**							
	Intensivist	16 (48)		11 (36)		27 (42)	
	Intensive care unit fellow	5 (15)		6 (19)		11 (17)	
	Resident^c^	12 (36)		14 (45)		26 (41)	
**Medical specialty**							
	Internal medicine	11 (33)		7 (23)		18 (28)	
	Anesthesiology	11 (33)		16 (52)		27 (42)	
	Pediatric medicine	3 (9)		0 (0)		3 (5)	
	Emergency medicine	1 (3)		0 (0)		1 (2)	
	Pulmonology	1 (3)		2 (7)		3 (5)	
	Surgery	1 (3)		0 (0)		1 (2)	
	Neurosurgery	1 (3)		0 (0)		1 (2)	
	Neurology	1 (3)		0 (0)		1 (2)	
	Resident not in training^c^	3 (9)		6 (16)		9 (14)	

^a^The response rate for this group was 33 of 40 (83%).

^b^The response rate for this group was 31 of 53 (58%).

^c^Includes physician assistants.

### Current Decision-Making Behavior With Respect to Discharging ICU Patients

**Figure 1:**
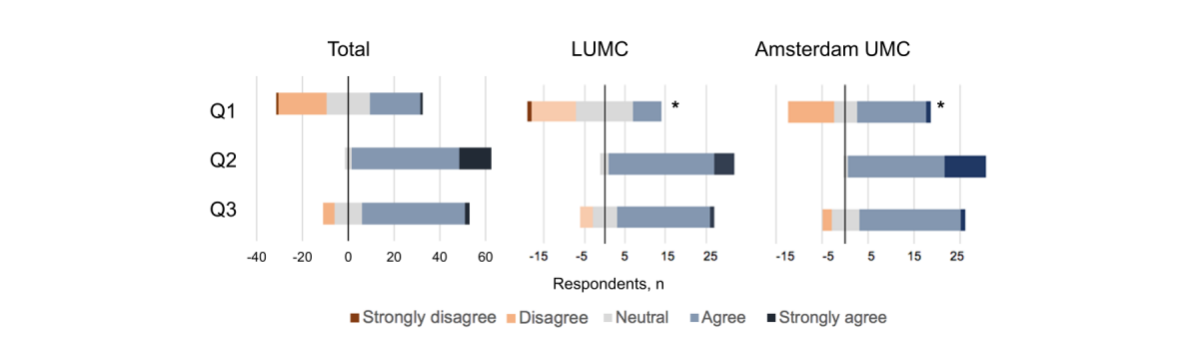
Responses to statements regarding current intensive care unit discharge practices. The bar width of the answers indicates the number of respondents that chose that option. Q1: “The decision to discharge a patient to a lower care ward is complex”; Q2: “A patient's ICU readmission risk is an important factor in my decision to discharge”; Q3: “I take bed availability into account for my decision to discharge a patient.” LUMC: Leiden University Medical Center; UMC: University Medical Center. *P<.05.

Responses on current discharge practices are visualized in Figure 1. Physicians disagreed on the complexity of the decision to discharge a patient from the ICU, with 23 of 64 (36%) agreeing or strongly agreeing with the Q1 statement and 22 of 64 (34%) disagreeing or strongly disagreeing (Table 2). A nonsignificant difference was observed between experienced intensivists and other physicians (Multimedia Appendix 3). For question 2, 61 of 64 (95%) of physicians agreed or strongly agreed that readmission was an important factor in the decision to discharge a patient. Besides a patient’s readmission risk, physicians indicated that bed availability was an important factor in their decision to discharge (47/64, 73%, Q3). Furthermore, we asked physicians to report their average certainty regarding their estimation of the readmission risk of a patient after discharge. The median certainty score was 7 (IQR 7-8) for the whole group, with no significant difference observed between the two locations (*P*=.79). Patient groups for which the decision to discharge was perceived to be most challenging included patients with a long length of ICU stay (44/64, 69%) and readmitted patients (44/64, 69%; Table 3). Multimedia Appendix 4 shows the open-answer questions regarding patient groups and clinically relevant patient factors in the decision to discharge. The most reported reason for a complex decision to discharge was case complexity (9/64, 14%). The most frequently mentioned factor influencing the decision to discharge a patient (in relation to the patient’s clinical state, process-related factors, and factors related to the receiving ward) was the level of care and facilities at the ward (23/64, 36%), followed by the general clinical state of the patient (17/64, 27%) and the patient’s alarm ability (10/64, 16%).

**Table 2:** Responses to statements. *P* values in italics represent a significant difference (*P*<.05) between the Leiden University Medical Center and Amsterdam University Medical Center respondents. Results are reported on a 5-point Likert scale, ranging from 1 (strongly disagree) to 5 (strongly agree). Scores >3 indicate median agreement with the statement and results <3 median disagreement.

Question			Total, median (IQR)		Leiden University Medical Center, median (IQR)		Amsterdam University Medical Center, median (IQR)		*P* values^a^
**Domain 1: Physicians’ current decision-making behavior with respect to discharging ICU^b^ patients**									
	Q1: “The decision to discharge a patient to a lower care ward is complex”	3 (2-4)		3 (2-3)		4 (2-4)		*.04*	
	Q2: “A patient’s ICU readmission risk is an important factor in my decision to discharge”	4 (4-4)		4 (4-4)		4 (4-5)		.09	
	Q3: “I take bed availability into account for my decision to discharge a patient”	4 (3-4)		4 (3-4)		4 (3.5-4)		.43	
**Domain 2: Physicians’ perspectives on the use of artificial intelligence (AI^c^)–based clinical decision support tools in general**									
	Q4: “I am familiar with the concept of AI”	4 (4-4.25)		4 (4-4)		4 (4-5)		*.004*	
	Q5: “I believe AI could support me in my work as physician”	4 (4-4)		4 (4-4)		4 (4-4.5)		*.006*	
	Q6: “I believe that AI will take over my job in the future”	2 (2-3)		2 (2-3)		2 (2-2.5)		.22	
	Q7: “I believe AI understands my work sufficiently in order to support me”	3 (3-4)		3 (3-4)		3 (3-4)		.39	
	Q8: “I believe in the added value of AI based decision support at the ICU”	4 (4-4)		4 (4-4)		4 (4-4)		.41	
**Domain 3: Physicians’ willingness to incorporate the discharge decision support tool in daily clinical practice**									
	Q9: “An AI based decision support for ICU readmission could be of positive value in the decision to discharge a patient”	4 (4-4)		4 (4-4)		4 (4-4)		*.02*	
	Q10: “It is important for me to have insight in the contributing factors to the predicted chance of readmission”	4 (4-4.25)		4 (4-5)		4 (4-4)		*.03*	
	Q18: “I assume that no readmission risk prediction score could influence my behavior”	2 (2-2)		2 (2-2)		2 (2-2)		.11	
	Q19: “I’m willing to consult the prediction of the decision support tool before making my decision to discharge a patient”	4 (4-4)		4 (4-4)		4 (4-4)		.47	
	Q20: “Taking into account the current workload at my department, I have time to take in the prediction score provided by the decision support tool and to take this into account for my decision to discharge a patient”	4 (4-4)		4 (3-4)		4 (4-4)		.11	

^a^*P* values were calculated with the Mann-Whitney *U* test.

^b^ICU: intensive care unit.

^c^AI: artificial intelligence.

**Table 3:** Patient groups for which the decision to discharge was perceived as most challenging (one or more options could be chosen).

Patient groups	Respondents, n (%)
Long admission	44 (69)
Currently readmitted	44 (69)
Elderly	7 (11)
COVID-19	4 (6)
Other	12 (19)

### Attitudes and Perspectives Toward AI-CDS Tools in the ICU

**Figure 2:**
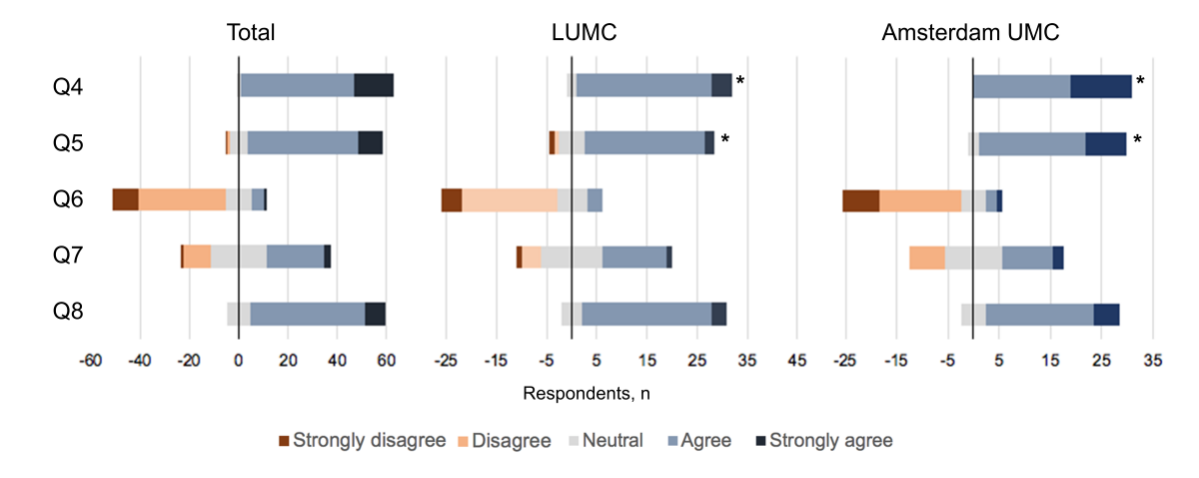
Statements regarding the attitude toward the use of artificial intelligence–based decision support tools in the intensive care unit. Q4: “I am familiar with the concept of AI”; Q5: “I believe AI could support me in my work as physician”; Q6: “I believe that AI will take over my job in the future”; Q7: “I believe AI understands my work sufficiently in order to support me”; Q8: “I believe in the added value of AI based decision support at the ICU.” LUMC: Leiden University Medical Center; UMC: University Medical Center. *P<.05.

The respondents were familiar with the concept of AI (62/64, 97% agreed or strongly agreed with Q4) and the majority agreed that AI could support them in their work as a physician (55/64, 86% agreed with Q5; Figure 2). Respondents from the development site (Amsterdam UMC) were more familiar with the concept of AI (*P*=.004, Q4) and agreed more with the statement that AI could support them in their work as a physician (*P*=.006, Q5) than the LUMC respondents. The majority did not believe that AI would take over their job in the future (46/64, 72% disagreed or strongly disagreed with Q6), and the respondents were indecisive on whether AI understood their work sufficiently to support them (26/64, 41% agreed or strongly agreed and 12/64, 19% disagreed or strongly disagreed with Q7). Nevertheless, 55 of 64 (86%) respondents believed in the added value of AI-CDS in the ICU (Q8). The more experienced intensivists agreed significantly less with the statement “I believe AI could support me in my work as a physician” (Multimedia Appendix 4). This finding was compatible with the responses to Q7 and Q8, indicating that the more experienced respondents were less convinced that AI understood their work sufficiently and that AI could be of added value at the ICU.

### Willingness to Incorporate a Discharge AI-CDS Tool in Daily Clinical Practice

**Figure 3:**
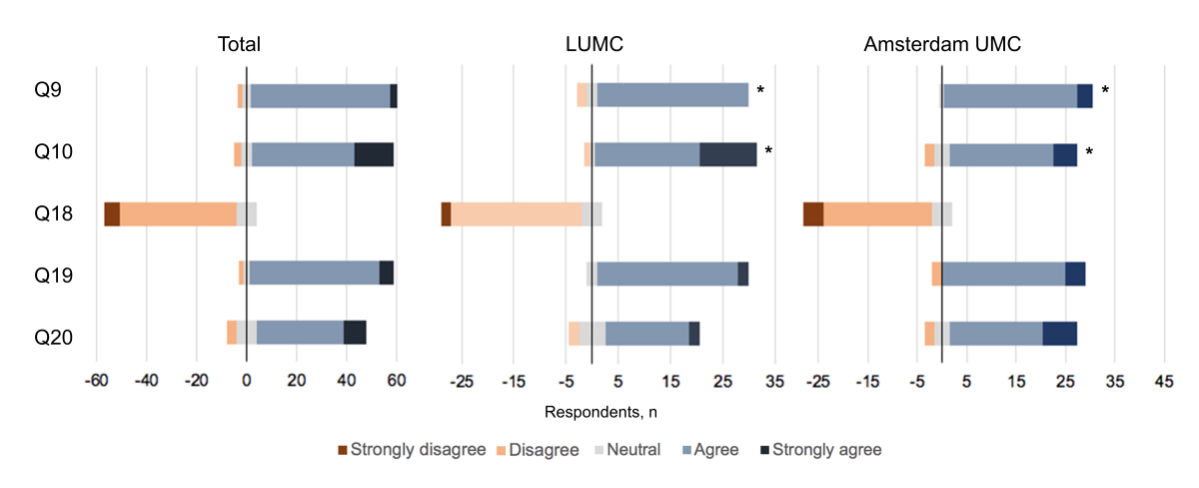
Statements regarding willingness to incorporate a discharge decision support tool in daily clinical practice. Q9: “An AI based decision support for ICU readmission could be of positive value in the decision to discharge a patient”; Q10: “It is important for me to have insight in the contributing factors to the predicted chance of readmission”; Q18: “I assume that no readmission risk prediction score could influence my behavior”; Q19: “I’m willing to consult the prediction of the decision support tool before making my decision to discharge a patient”; Q20: “Taking into account the current workload at my department, I have time to take in the prediction score provided by the decision support tool and to take this into account for my decision to discharge a patient.” LUMC: Leiden University Medical Center; UMC: University Medical Center. *P<.05.

The respondents agreed or strongly agreed that a discharge AI-CDS tool could be of positive value (59/64, 92%; Q9), and were willing to take the time to consult the AI-CDS and to take the prediction of the tool into consideration before discharging a patient (44/64, 69%; Q20 and 58/64, 91%; Q19; Figure 3). Furthermore, respondents disagreed or strongly disagreed with the statement “I assume that no readmission risk prediction score could influence my behavior” (53/64, 83%, Q18). Amsterdam UMC respondents agreed more with the statement “an AI based decision support for ICU readmission could be of positive value in the decision to discharge a patient” than the LUMC respondents (*P*=.02; Q9), but the difference was small. Q10 emphasizes the need of physicians for prediction tools to be explainable (57/64, 89% agreed or strongly agreed). This need was more important for the LUMC physicians (*P*=.03).

**Figure 4:**
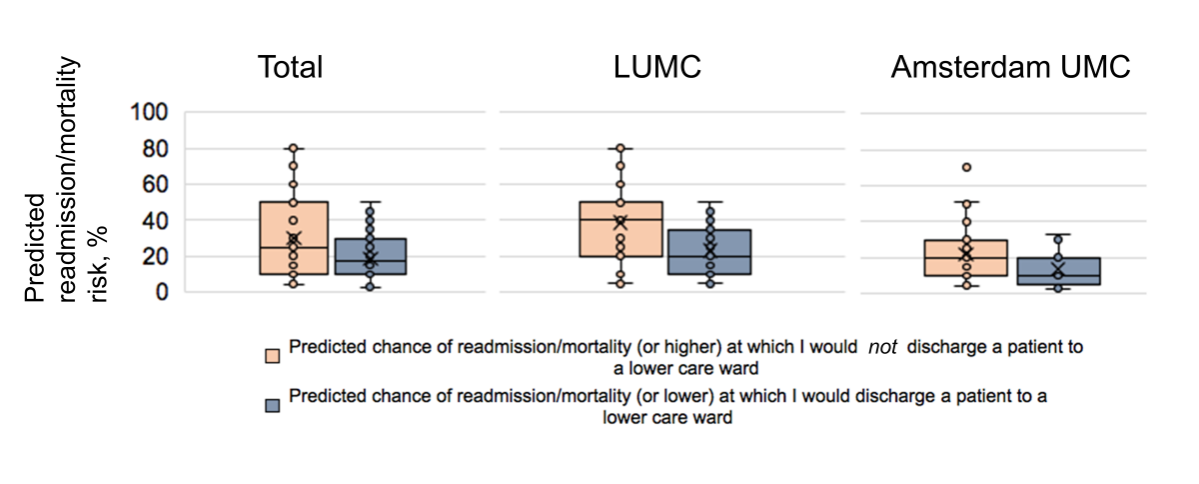
Predicted readmission and mortality risk that would influence physicians’ behavior in discharging or not discharging an intensive care unit patient. LUMC: Leiden University Medical Center; UMC: University Medical Center.

Physicians were asked to indicate the threshold of predicted readmission and mortality risk (on a scale from 0 to 100) above which they would not discharge a patient to the ward, and the threshold below which they would discharge a patient (Figure 4). Results varied widely. The LUMC respondents reported that a median 40% (IQR 20%-50%) readmission and mortality risk or higher would cause them to postpone discharge, compared to a 20% (IQR 10%-30%) risk for the Amsterdam UMC group. The LUMC group indicated that a median readmission and mortality risk of 20% (IQR 10%-33%) or lower would be acceptable to discharge a patient, compared to a 10% (IQR 7.5%-20%) risk for the Amsterdam UMC group.

### Desired Workflow for the Tool and End Users

A total of 40 of 64 (63%) of the ICU physicians preferred that risk prediction be integrated in EHRs, while 21 of 64 (33%) preferred a stand-alone dashboard. The moments that the respondents most often chose for the AI-CDS to be displayed were during morning handover (24/64, 38%), morning rounds (21/64, 33%), and grand rounds or bedside multidisciplinary consultations (28/65, 44%; Table 4). The respondents indicated that AI-CDS predictions, if they were not continuous, should be updated before these moments to be of value to the end users. The tool was indicated to be most relevant for supervisors (ie, responsible board-certified intensivists; 62/64, 97%), intensive care fellows (57/64, 89%) and residents (42/64, 66%; Table 5).

**Table 4:** Desired moment to display the prediction tool, with approximate times. More than one option could be chosen.

Moments	Time	Respondents (N=64), n (%)
Morning handover	7:45 AM	24 (38)
Before morning rounds	8:30 AM	6 (9)
Morning rounds	8:45 AM	21 (33)
Grand rounds or bedside multidisciplinary consultation	11:30 AM	28 (44)
Multidisciplinary consultation	2 PM	17 (27)
Evening rounds	4:15 PM	6 (9)
Daily care	All day	8 (13)

**Table 5:** End users for whom the tool was deemed to be most relevant. More than one option could be chosen.

End users	Respondents (N=64), n (%)
Bed coordinators	33 (52)
Supervisors, intensive care physicians	62 (97)
Intensive care fellows	57 (89)
Residents	44 (66)
Nurses	34 (53)

### Open Comments

At the end of the survey, physicians could leave open comments and suggestions. Two physicians indicated a need for insights into what patient factors the predictions were based on to consider the tool safe and trustworthy. Besides the need for model explainability, a need for further validation of the tool before being able to trust it was mentioned. Furthermore, the combined outcome prediction (ie, readmission or mortality) was found to be problematic, with one physician expressing willingness to accept a high risk of readmission, but not mortality. Another comment was related to the finding that bed availability was important in the decision to discharge, as multiple physicians mentioned that they would accept a high risk of readmission if the decision freed a bed for a liver-transplant patient, for example.

## Discussion

This study assessed the preimplementation of AI-CDS tools across 4 domains: physicians’ current decision-making behavior regarding ICU discharge, their perspectives on AI, and their preferences for an AI-CDS tool’s implementation and use in clinical practice. We found that nearly all ICU physicians were familiar with AI and had positive expectations, with 55 of 64 (86%) believing that AI could support them in their work as physicians. Not all physicians found the decision to discharge a patient complex, yet 59 of 64 (92%) agreed that a discharge decision support tool could be of value. Physicians at the site where the AI-CDS tool was developed showed greater familiarity with AI and had a stronger belief in the supportive role of AI in general, but also had a stronger belief that an AI-CDS tool specifically for discharge decision support would be useful compared to physicians at the nondevelopment site. Physicians from the nondevelopment site attached more importance to understanding which factors contributed to the predictions.

A positive attitude among physicians toward the use of the AI-CDS tool has also been found in other studies [15,16,18,20]. Interestingly, most respondents in our study believed in the added value of AI-CDS tools, while only 26 of 64 (41%) agreed or strongly agreed that AI understood their work sufficiently to support them. As in previous surveys [18,20], this incongruous finding could be explained by the fact that these physicians had not worked with AI-CDS tools when the study was conducted, and they therefore did not know if these tools were capable of capturing the complex ICU environment [28]. Lastly, the literature confirms the effect of bed capacity on physicians’ decision to discharge, which could limit the applicability of the AI-CDS tool in settings where bed capacity is low [21].

A recent scoping review of guidelines for the development of AI-CDS tools concluded that more focus on implementation strategy is needed for effective integration in the clinical setting [29]. Human-factors research, in the form of qualitative interviews and questionnaires, may enhance the uptake of AI-CDS tools, as this approach may improve the system’s design, training process, and implementation strategies [12,17]. We recommend focusing on the important local and sociotechnical context of each preimplementation site to meet the challenge of the “last mile” of implementation [11,21,22,30,31]. The positive attitudes and willingness to use AI-CDS tools we observed are positive indicators of the acceptance of this new technology [32,33], but they also underpin the idea that expectations should be aligned with the intended use of the AI-CDS tool to be adopted [17]. Moreover, it will be of value to repeat our questionnaire after the implementation of the AI-CDS tool for discharging ICU patients, as it has previously been observed that physicians showed reduced excitement (*P*<.01) about AI-CDS after implementation [34].

As illustrated by the differences in familiarity and enthusiasm toward AI-CDS at the development and nondevelopment sites, sufficient attention should be paid to training and informing physicians on the use of the AI-CDS tool in their daily practice [10]. This training should also encompass the ethics and responsibilities of using AI-CDS in health care, as the physicians retain final responsibility for treatment decisions [33]. Lastly, training will be needed to educate physicians on the interpretation of the mortality or readmission risk predictions, as we observed a range of answers regarding the threshold at which patients would or would not be discharged to lower care wards (Figure 3). Due to a significant imbalance in the number of patients that were or were not readmitted or died after discharge, risk predictions are skewed along the 0% to 100% scale, being concentrated around an event rate of 5.3% [25]; the respondents were not informed of this. Therefore, attention should be paid to the interpretation of these calibrated risk predictions during training, as perceptions clearly differed on what constituted high and low risks for this outcome.

The implications of this study for the design process of AI-CDS tools include the need for explainable AI, as most respondents indicated a need to have insight into the factors contributing to “black box” predictions. We want to stress that addressing explainability is not the only factor required for a successful AI-CDS implementation; rather, the incorporation of domain expertise, the sociotechnical context, and physicians’ perspectives should be taken into account during the whole development, design, and implementation process [31,35,36]. We recommend that AI-CDS developers perform user and human-factor research in an early phase of design and development to maximize impact and smooth integration into the current decision-making process [11].

A limitation of the current study was that we only conducted the survey at 2 academic tertiary referral hospitals in the Netherlands. This could reduce the generalizability of our findings; for example, ICU physicians from nonacademic hospitals may be less familiar with AI. Secondly, the respondents may have had differences in their understanding of AI, as we did not provide a clear definition of AI to the end users in order to keep the questionnaire concise. Another limitation was that we did not formally assess the validity and reliability of this questionnaire. However, we did construct the questionnaire with a broad team of experts and performed a feasibility study at LUMC before generalizing the questions to be applicable to Amsterdam UMC. Future research could develop validated questionnaires for the preimplementation of AI-CDS tools, and the 4 domains presented here relating to current decision-making, workflow, and perspectives toward AI-CDS may serve as a blueprint. The increased workload caused by the pandemic may have impacted our response rate (64/93; 69%). However, the different levels of training and variety of medical specialties of physicians working at ICUs were represented in our sample of ICU clinicians, and few differences were observed between experienced ICU physicians and other respondents (Multimedia Appendix 3). Nevertheless, a nonresponse bias may have affected our results, as the clinicians that did fill in the questionnaire could have had a higher interest in AI-CDS compared to nonrespondents.

To conclude, this survey provides valuable insights into current decision-making behavior and perspectives on the use of AI-CDS tools that can be used in the implementation process and the training of end users. Positive attitudes were reported toward AI-CDS in general and for an AI-CDS tool for discharging ICU patients in particular, even though not all the respondents perceived the decision to discharge a patient to be complex. Observed differences between the 2 study sites, which had different levels of involvement in the development of the AI-CDS tool, show the need for education and training in departments with little experience with AI-CDS. We recommend that developers of AI-CDS tools involve their end users early in the design process and perform preimplementation by means of surveys to investigate potential acceptance in the local context, improve the system’s design and clinical workflow design, and ultimately facilitate clinical uptake.

## Appendix

Multimedia Appendix 1 Rationale per question in the questionnaire.

Multimedia Appendix 2 Original questionnaire.

Multimedia Appendix 3 Statement questions: intensivists versus other participants.

Multimedia Appendix 4 Open answer questions.

## Abbreviations

AI: artificial intelligence
AI-CDS: artificial intelligence–based clinical decision support
EHR: electronic health record
ICU: intensive care unit
LUMC: Leiden University Medical Center
UMC: University Medical Center
