# Supplementary material for: Intensive Care Unit Physicians’ Perspectives on Artificial Intelligence–Based Clinical Decision Support Tools: Preimplementation Survey Study
Source: JMIR Hum Factors. 2023 Jan 5;10:e39114. doi: 10.2196/39114 (PMC9853335; doi:10.2196/39114)
Supplement: Multimedia Appendix 1 [file humanfactors_v10i1e39114_app1.docx]

**Intensive Care Unit Physicians’ Perspectives on Artificial Intelligence-Based Clinical Decision Support Tools: Preimplementation Survey Study**

S.L. van der Meijden, A.A.H. de Hond, P.J. Thoral, I.M.J. Kant, E.W. Steyerberg, G. Cina, M.S. Arbous

**Multimedia Appendix 1: Rationale per question in the questionnaire**

Table 1-1: Rationale behind each survey item

| **Domain** | **Question** | **Rationale** |
| --- | --- | --- |
| 1 - Physicians’ current decision-making behavior with respect to discharging ICU patients | Q1 - The decision to discharge a patient to a lower care ward is complex | To determine whether the decision to discharge ICU patient’s is perceived as complex and therefore could benefit from decision support tools |
|  | Q2 - A patient's ICU readmission risk is an important factor in my decision to discharge | To determine whether the predicted readmission risk (which is the predicted outcome of the AI-CDS tool in question) is an important factor in the current way of working |
|  | Q3 - I take bed availability into account for my decision to discharge a patient | To determine whether this not-patient related factor influences discharge decision making |
| 2 - Perspectives on the use of AI-CDS tools in general | Q4 - I am familiar with the concept of AI | To determine whether the participants feel like they are familiar with this technology that has not been implemented in the study sites |
|  | Q5 - I believe AI could support me in my work as physician | To determine the pre-implementation believe that an AI tool could be of support |
|  | Q6 - I believe that AI will take over my job in the future | To uncover whether ICU clinicians see AI as a treath to their job |
|  | Q7 - I believe AI understands my work sufficiently in order to support me | To uncover whether the participants believe that AI is able to grasp the complexity of their work and therefore be of support |
|  | Q8 - I believe in the added value of AI based decision support at the ICU | To determine pre-implementation whether an AI tool could be of support in the ICU setting |
| 3 - Willingness to incorporate the discharge AI-CDS tool into daily clinical practice | Q9 - An AI based decision support for ICU readmission could be of positive value in the decision to discharge a patient | To determine pre-implementation whether the participants believe in the added value of a discharge decision support tool |
|  | Q10 - It is important for me to have insight in the contributing factors to the predicted chance of readmission | To determine the need to communicate or vizualize the underlying predictors of the (‘black-box’) AI algorithms |
| 1 - Physicians’ current decision-making behavior with respect to discharging ICU patients | Q11 - How certain are you in general that a patient will not be readmitted to the ICU after your decision to discharge a patient? (Patients with a no-return policy excluded) | To determine what level of uncertainty the participants allow when they discharge an ICU patient |
|  | Q12 - I find the decision/timing to discharge a patient from the ICU most challenging for the following group(s) (you may choose more than one answer):   - Long admitted ICU patients: …> days - Previously readmitted patients - Patients above age … years - COVID patients - Other; ….. | To uncover for which patient groups the discharge AI-CDS tool would be of value to the participants, and for whom ideally the tool should have an accurate performance |
|  | Q13 - Which factors do you find most important in the discharge process? | To get an understanding of the clinical and non-clinical factors that are related to the process the AI-CDS tool in question is aiming to address |
| 4 - Preferences for using a discharge AI-CDS tool in daily workflows | Q14 - The predicted readmission/mortality probability per patient should be displayed during (you may choose more than one answer):   - The morning handover - Before morning rounds - Morning rounds - Grand rounds/bed-side multidisciplinary consultation - Multidisciplinary consultation - Evening rounds - Daily care | To determine for which moments in the daily clinical workflow the AI-CDS tool should be accessible and updated. Furthermore, to understand the demands on the user interface by knowing the time and environments the tool would be most often accessed. Particularly as the tool in its current version is intended to be used on a web application and is not yet suitable for mobile devices Furthermore, knowledge of the exact time and location the tool is intended to be used dictates training activities. And finally, to increase adherence to the use of the AI-CDS, the use of the AI-CDS tool should be preferably incorporated into the workflow at the moments that are seen as most valuable. |
|  | Q15 - The place of preference of displaying the predicted readmission/mortality probability would be:   - Tab ‘overview’ in the EHR - Tab ‘status’ in the EHR - As a separate dashboard in the EHR - Other … | To determine what place in the EHR the prediction should be shown and implemented |
|  | Q16 - The discharge decision support tool for ICU patients would be most relevant for (you may choose more than one answer):   - Bed coordinator - Supervisor/intensive care physician - Fellow intensive care - Resident - Nurse | To determine the potential intended users for the AI-CDS tool and for whom the tool will be of most value |
| 3 - Willingness to incorporate the discharge AI-CDS tool into daily clinical practice | Q17 - Above/below what threshold of predicted readmission/mortality risk (on a scale between 0 and 100) would you (not) discharge an ICU patient to the ward? | To study the potential influence of a certain predicted chance of readmission/mortality on the physician's behavior |
|  | Q18 - I assume that no readmission risk prediction score could influence my behavior | To uncover pre-implementation whether participants assume that they will not let their decision-making be influenced by the AI-CDS |
|  | Q19 - I'm willing to consult the prediction of the decision support tool before making my decision to discharge a patient | To uncover pre-implementation whether participants are willing to take the AI-CDS prediction into account in the process of decision making to discharge an ICU patient |
|  | Q20 - Taking into account the current workload at my department, I have time to take in the prediction score provided by the decision support tool and to take this into account for my decision to discharge a patient | To uncover pre-implementation whether participants perceive that they have enough time to consult an AI-CDS tool before discharging an ICU patient |
