# Supplementary material for: Intensive Care Unit Physicians’ Perspectives on Artificial Intelligence–Based Clinical Decision Support Tools: Preimplementation Survey Study
Source: JMIR Hum Factors. 2023 Jan 5;10:e39114. doi: 10.2196/39114 (PMC9853335; doi:10.2196/39114)
Supplement: Multimedia Appendix 2 [file humanfactors_v10i1e39114_app2.docx]

**Intensive Care Unit Physicians’ Perspectives on Artificial Intelligence-Based Clinical Decision Support Tools: Preimplementation Survey Study**

S.L. van der Meijden, A.A.H. de Hond, P.J. Thoral, I.M.J. Kant, E.W. Steyerberg, G. Cina, M.S. Arbous

**Multimedia Appendix 2: Original questionnaire (in Dutch)**

**
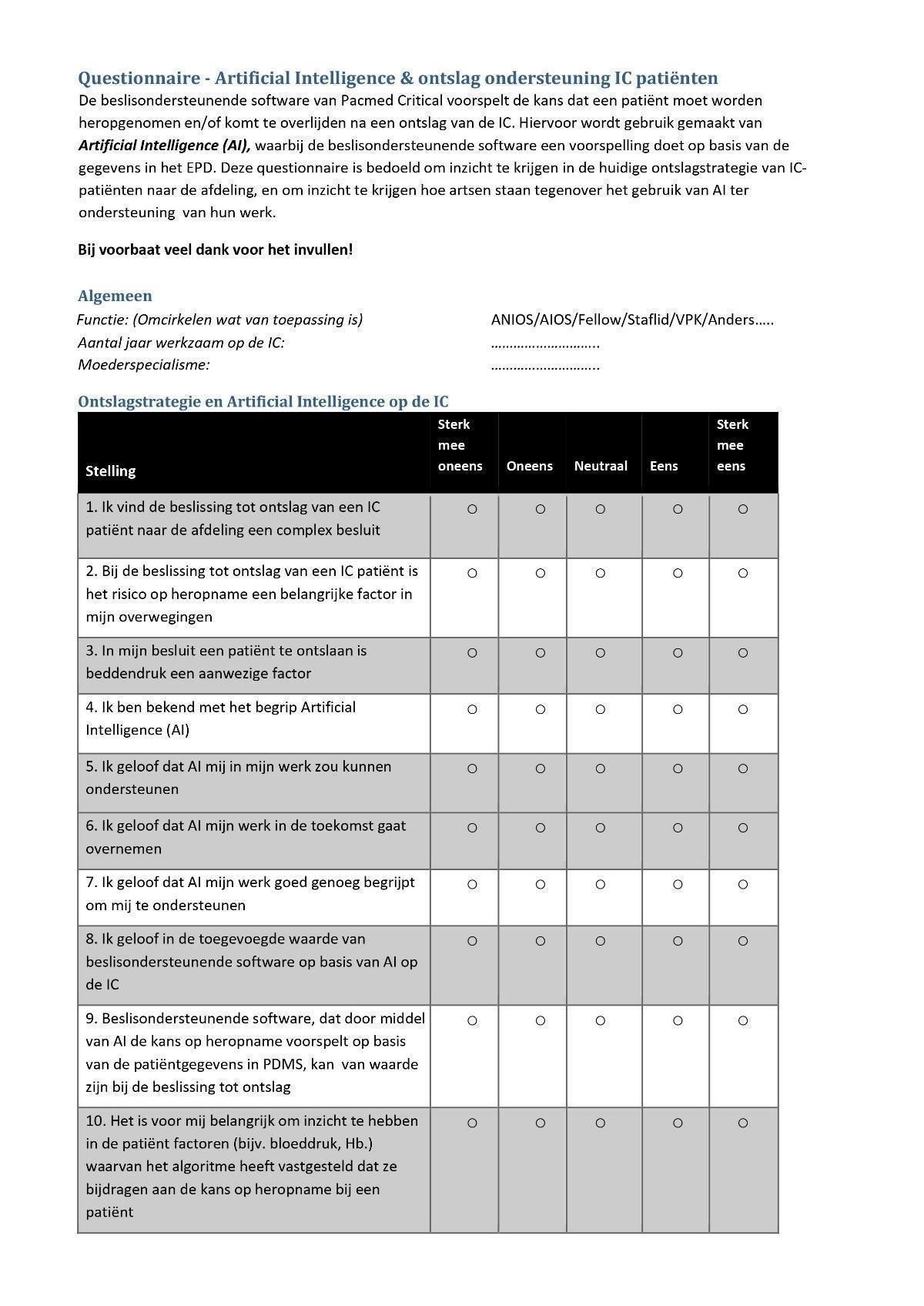
**

**
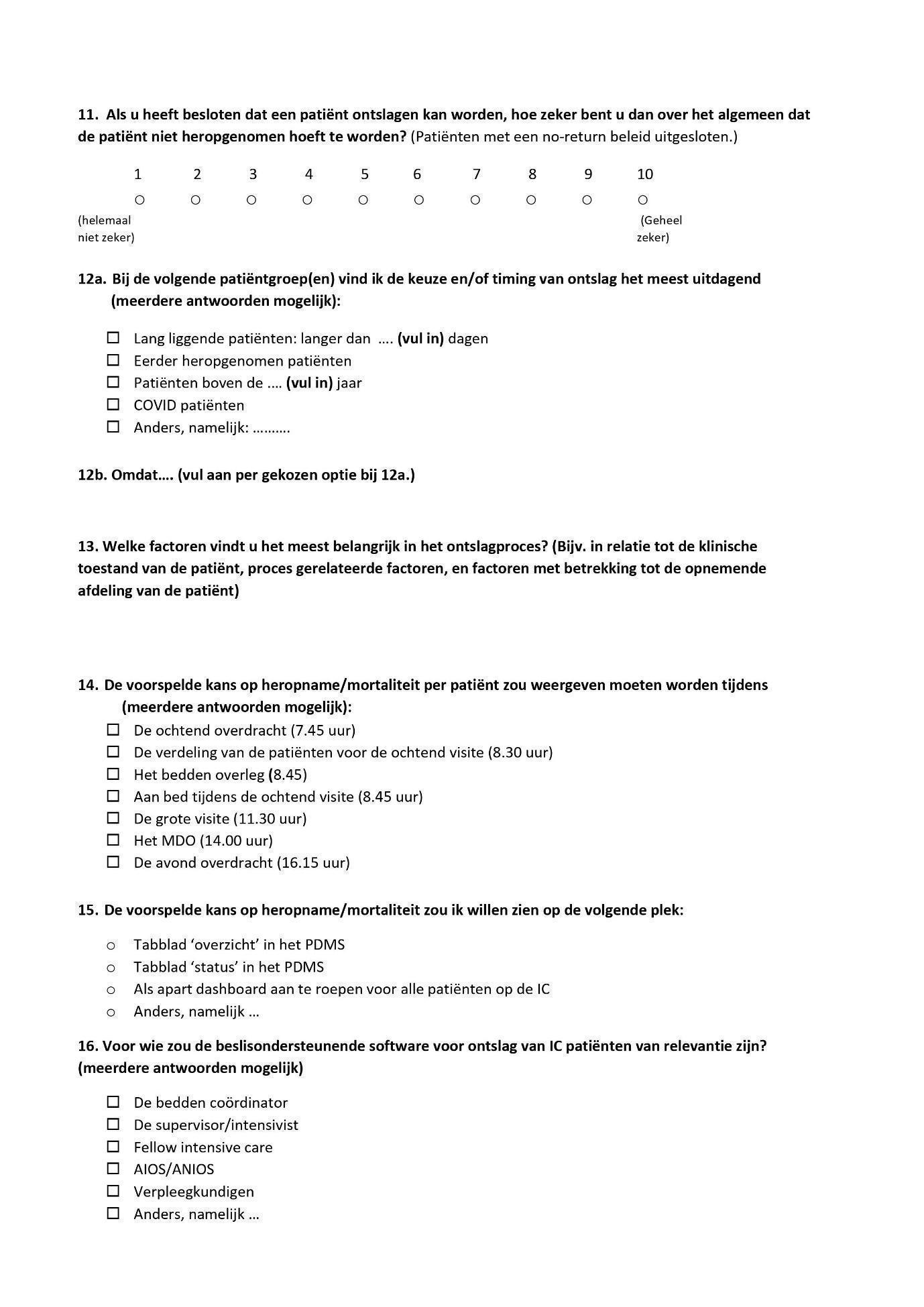
**

**
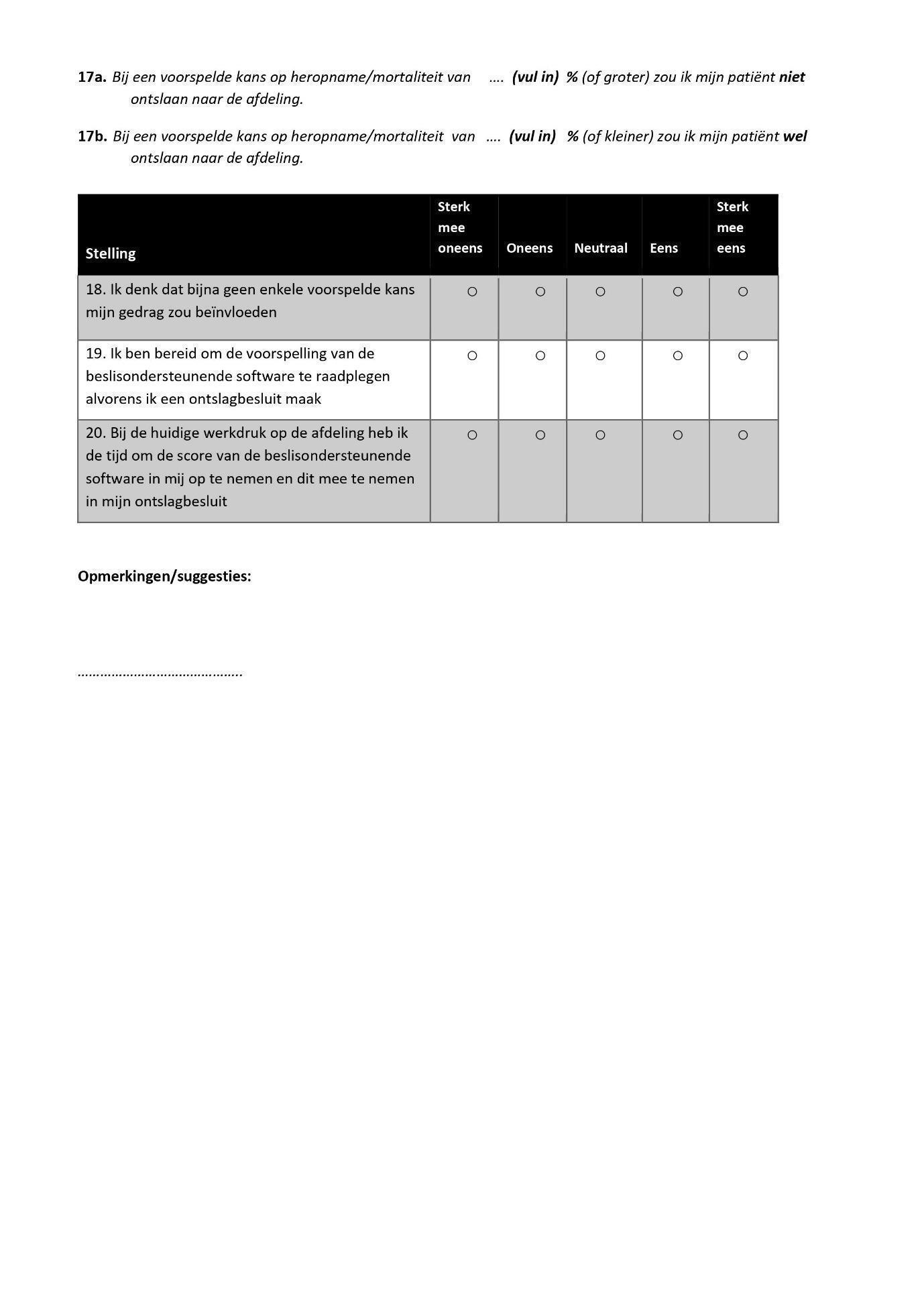
**
