# Supplementary material for: Intensive Care Unit Physicians’ Perspectives on Artificial Intelligence–Based Clinical Decision Support Tools: Preimplementation Survey Study
Source: JMIR Hum Factors. 2023 Jan 5;10:e39114. doi: 10.2196/39114 (PMC9853335; doi:10.2196/39114)
Supplement: Multimedia Appendix 3 [file humanfactors_v10i1e39114_app3.docx]

**Intensive Care Unit Physicians’ Perspectives on Artificial Intelligence-Based Clinical Decision Support Tools: Preimplementation Survey Study**

S.L. van der Meijden, A.A.H. de Hond, P.J. Thoral, I.M.J. Kant, E.W. Steyerberg, G. Cina, M.S. Arbous

**Multimedia Appendix 3: Statement questions: intensivists versus other participants**

*Table 3-1: Statement question results. IQR = Interquartile Range. P-values in italics represent a significant difference between intensivists and other ICU physicians (P<.05). Results are reported on a five-point Likert Scale, ranging from 1 (strongly disagree) to 5 (strongly agree). Results > 3.0 indicate median agreement and results < 3.0 median disagreement.*

| Question | Total median (IQR) | Intensivists (IQR) | Other respondents (IQR) | *P*-values^a^ |
| --- | --- | --- | --- | --- |
| Q1 - The decision to discharge a patient to a lower care ward is complex | 3 (2 - 4) | 3 (3 - 4) | 3 (2 - 4) | 0.16 |
| Q2 - A patient's ICU readmission risk is an important factor in my decision to discharge | 4 (4 - 4) | 4 (4 - 5) | 4 (4 - 4) | 0.09 |
| Q3 - I take bed availability into account for my decision to discharge a patient | 4 (3 - 4) | 4 (4 - 4) | 4 (3 - 4) | 0.10 |
| Q4 - I am familiar with the concept of AI | 4 (4 - 4.25) | 4 (4 - 4) | 4 (4 - 5) | 0.13 |
| Q5 - I believe AI could support me in my work as physician | 4 (4 - 4) | 4 (4 - 4) | 4 (4 - 4) | *0.006* |
| Q6 - I believe that AI will take over my job in the future | 2 (2 - 3) | 2 (2 - 2.5) | 2 (2 - 3) | 0.47 |
| Q7 - I believe AI understands my work sufficiently in order to support me | 3 (3 - 4) | 3 (3 - 3.5) | 4 (3 - 4) | *0.018* |
| Q8 - I believe in the added value of AI based decision support at the ICU | 4 (4 - 4) | 4 (3.5 - 4) | 4 (4 - 4) | *0.013* |
| Q9 - An AI based decision support for ICU readmission could be of positive value in the decision to discharge a patient | 4 (4 - 4) | 4 (4 - 4) | 4 (4 - 4) | 0.06 |
| Q10 - It is important for me to have insight in the contributing factors to the predicted chance of readmission | 4 (4 - 4.25) | 4 (4 - 4) | 4 (4 - 5) | 0.39 |
| Q18 - I assume that no readmission risk prediction score could influence my behavior | 2 (2 - 2) | 2 (2 - 2) | 2 (2 - 2) | 0.11 |
| Q19 - I'm willing to consult the prediction of the decision support tool before making my decision to discharge a patient | 4 (4 - 4) | 4 (4 - 4) | 4 (4 - 4) | 0.33 |
| Q20 - Taking into account the current workload at my department, I have time to take in the prediction score provided by the decision support tool and to take this into account for my decision to discharge a patient | 4 (4 - 4) | 4 (4 - 4) | 4 (4 - 4) | 0.40 |

^a^P-Values were calculated with the Mann-Whitney U test.
Orange questions (Q1-Q3) belong to the domain of investigating physicians’ current decision-making behavior with respect to discharging ICU patients.
Blue questions (Q4-Q8) belong to the domain of physicians’ perspectives on the use of AI decision support tools in general.
Red questions (Q9-Q20) belong to the domain of physicians’ willingness to incorporate the tool discharge decision support tool in daily clinical practice.
