# Supplementary material for: Intensive Care Unit Physicians’ Perspectives on Artificial Intelligence–Based Clinical Decision Support Tools: Preimplementation Survey Study
Source: JMIR Hum Factors. 2023 Jan 5;10:e39114. doi: 10.2196/39114 (PMC9853335; doi:10.2196/39114)
Supplement: Multimedia Appendix 4 [file humanfactors_v10i1e39114_app4.docx]

**Intensive Care Unit Physicians’ Perspectives on Artificial Intelligence-Based Clinical Decision Support Tools: Preimplementation Survey Study**

S.L. van der Meijden, A.A.H. de Hond, P.J. Thoral, I.M.J. Kant, E.W. Steyerberg, G. Cina, M.S. Arbous

**Multimedia Appendix 4: Open answer questions**

**Q12b: Reasons why discharge is perceived as complex for certain patient groups.**

Total number of answers: n = 33 (51.6%)

- Complex cases (including comorbidities and complications) (n = 9)
- Patients are too weak for the ward (n = 5)
- Risk of repeated readmission (n = 5)
- Reduced intensity of care at ward (n = 4)
- Reduced coughing strength and other respiratory problems (n = 4)
- Vulnerable patients (n = 2)
- ICU-acquired weakness (n = 2)
- Infection risk (n = 1)
- Patient not adequate enough to alarm themselves (n = 1)

**Q13: What factors are most important in your decision to discharge a patient from the ICU?**

Total number of answers: 61 respondents (95.3%) answered one or more factors.

1. Factors related to receiving ward, daily care and logistics
   - Level of care and facilities at the ward (n = 23)
   - Burden of care (n = 5)
   - Good handover to ward (n = 3)
   - Bed availability (n = 2)
   - Nurses opinion on discharge readiness (n = 2)
   - Clear diagnosis and treatment plan at discharge (n = 1)
   - Patient’s length of stay at the ICU (n = 1)
2. Factors related to clinical state of a patient at moment of discharge
   - General clinical state of a patient (n = 17)
   - Patient need to be able to alarm (n = 10)
   - General strength of the patient (n = 8)
   - Neurology/cognitive status (n = 8)
   - Level of respiratory care needed (n = 5)
   - Stable vital functions (n = 5)
   - Respiratory status and coughing strength (n = 3)
   - Hemodynamic stability, low lactate, good SvO2 (n = 2)
   - Case complexity (n = 2)
   - Patient should be mobile (n = 1)
   - No longer in need of ICU support/monitoring (n = 1)
   - Risk of mortality (n = 1)
   - Risk of readmission (n = 1)
   - > 12 hours no longer in need of ICU related care (n = 1)
   - Stable situation for long admitted patients (n = 1)
   - Blood pressure (n = 1)
   - ICU acquired weakness (n = 1)
3. Factors related to the general condition of the patient
   - Comorbidities (n = 2)
   - Patient history (n = 1)
   - Pre-admission performance status (n = 1)
   - Chronic diseases (n = 1)
